# Multiple Sclerosis: Focus on Extracellular and Artificial Vesicles, Nanoparticles as Potential Therapeutic Approaches

**DOI:** 10.3390/ijms22168866

**Published:** 2021-08-18

**Authors:** Domenico Nuzzo, Pasquale Picone

**Affiliations:** 1 Istituto per la Ricerca e l’Innovazione Biomedica, CNR, via U. La Malfa 153, 90146 Palermo, Italy; domenico.nuzzo@cnr.it; 2 Dipartimento di Scienze e Tecnologie Biologiche Chimiche e Farmaceutiche, Università di Palermo, Viale delle Scienze, 90128 Palermo, Italy

**Keywords:** inflammation, nanotechnology, immunotolerance, drug delivery system

## Abstract

Multiple sclerosis (MS) is an autoimmune disease of the Central Nervous System, characterized by an inflammatory process leading to the destruction of myelin with neuronal death and neurodegeneration. In MS, lymphocytes cross the blood-brain barrier, creating inflammatory demyelinated plaques located primarily in the white matter. MS potential treatments involve various mechanisms of action on immune cells, immunosuppression, inhibition of the passage through the blood-brain barrier, and immunotolerance. Bio-nanotechnology represents a promising approach to improve the treatment of autoimmune diseases by its ability to affect the immune responses. The use of nanotechnology has been actively investigated for the development of new MS therapies. In this review, we summarize the results of the studies on natural and artificial vesicles and nanoparticles, and take a look to the future clinical perspectives for their application in the MS therapy.

## 1. Introduction

Multiple sclerosis (MS) is a chronic inflammatory disease of the Central Nervous System (CNS) [1,2], caused by a response of T and B cells towards the myelin sheath surrounding the axons of the CNS [3,4]. This process triggers an early inflammatory state, which ultimately culminates in demyelination and neuronal degeneration [2,5]. The origin of MS is still unknown. It is assumed that genetic and environmental factors (smoking, toxins), low vitamin D levels, obesity, microbial and viral infections, can contribute to its onset [1]. Worldwide, 2.3 million people are affected by MS, with an incidence rate of approximately 50–300 per 100,000 people [6]. MS can evolve into different profiles, Relapse-Remitting (RR-MS), in most patients (80–85% of MS cases) and, less common, primary progressive course (PP-MS) [7]. RR-MS can turn into a progressive course, referred to as secondary progressive (SP-MS) [7]. The typical neurological symptoms of MS include visual field disturbance, double and blurred vision, motor symptoms such as weakness of the face and extremities, bladder/bowel problems, walking difficulties, spasticity (stiffness and muscle spasms), and dizziness. The diagnosis of multiple sclerosis is based on the integration of clinical, imaging, and laboratory findings [7].

Treatment “first-line therapies” are mainly aimed at preventing the onset or relapse of patients. The administration of anti-inflammatory drugs is the most common therapy; attempts are made to extensively suppress the immune system, leading to the deletion or inactivation of entire subgroups of T cells [8].

In addition, to MS immunosuppressive treatments, there are other mechanisms of action on immune cells aimed to inhibit their passage through the Blood-Brain Barrier (BBB) and their immunotolerance. Interferon (IFN) administration, as IFN beta-1α and IFN beta-1β, has generally been used as “first-line therapy” [8], injected either intramuscularly or subcutaneously. IFN-beta 1α and 1β modulate T and B cells and regulate cytokine release [9]; however, these treatments exhibit a series of adverse effects and are only moderately efficacious. Natalizumab (the first approved intravenous drug) is a monoclonal antibody against integrin α4, which blocks the entry of lymphocytes into the CNS [10]. Although the efficiency of Natalizumab in MS relapse is superior to that of IFN, its administration is accompanied by noxious side effects [10]. Therefore, it is used in MS patients who do not respond positively to other treatments. Rituximab and Obinutuzumabis are antibodies against CD20 on B-lymphocytes, causing their depletion. Mitoxantrone has cytotoxic effects against B cells as well as T-helper and T-cytotoxic lymphocytes, but its use is limited due the side effects. In addition, several approaches aimed at restoring immune tolerance were tested [11]. Indeed, it is thought that the first event occurring in the MS pathogenesis is a tolerance breakdown, which determines the activation of naive myelin-specific T cells in healthy individuals [11]. Antigen-specific immunotherapies aim to restore immune tolerance without suppressing overall immune surveillance against microbes and cancer [11]. Previous studies with myelinated peptides (Antigens-Ags) in MS have shown that they are safe and that the peptides are capable of inducing immunomodulatory responses through several mechanisms [11]. The mechanisms underlying immune tolerance in MS were studied in animal models for three CNS myelin sheath proteins (proteolipid protein (PLP), myelin oligodendrocyte glycoprotein (MOG), and myelin basic protein (MBP) functioning as target autoantigens in experimental CD4 + T cell-mediated autoimmune encephalomyelitis (Experimental Autoimmune Encephalomyelitis–EAE) [11]. Full-length protein or fragments were used to induce an immune tolerance effect. Glatiramer Acetate (GA, Copaxone) [12] is a self-injectable drug composed of a copolymer of four amino acids within the MBP sequence that is recognized by reactive T lymphocytes. Administration of GA reduces the relapse rate and disease progression. GA prevents the binding of T cells to MBP and other myelin antigens, leading to the induction of tolerance. Furthermore, GA induces Th2 and T regulatory (Treg) lymphocytes and decreases the number of Th17 cells [12]. The previously considered MS therapies are administered systematically and therefore a significant amount of the drugs circulate in the bloodstream, interacting with non-target cells and causing adverse effects. Therefore, the selective and almost exclusive release of the drug to the action site would lead to considerable therapeutic advantage. Moreover, since MS usually affects the CNS, the way to effectively deliver the therapeutic agents from the bloodstream across the BBB is still a great challenge.

In the present review, we would like to focus on the recent advances in the development of nano-therapeutic approaches for the treatment of MS. Recently, natural or artificial nanovesicles and nanoparticles have been explored as potential approaches for MS treatment. A full understanding of the morphological, structural, and biological characteristics of natural vesicles and nanoparticles is important and gives the possibility to modify them for MS applications. Moreover, today, biotechnological techniques can lead to the generation of artificial vesicles that exhibit some or many of the features found in natural vesicles and cells, including membrane complexity/heterogeneity, specific interactions and/or compartmentalization.

## 2. Why Is a Nano-Therapeutic Approach Needed to Treat MS?

Multiple sclerosis is a complex disease, and its underlying mechanisms are only partially understood. Moreover, the involvement not only of peripheral immune cells (T and B lymphocytes) but also of the central ones (microglia) makes the therapeutic target complex. Therefore, in addition to acting at the peripheral level, the treatment must also act at the level of the CNS. Here, MS therapy is accompanied by low efficacy due to the presence of BBB and the occurrence of side effects due to the dispersion of the drugs that fail to enter the CNS [13]. Furthermore, it is important to administer therapeutic agents to the MS lesions present in different place of the CNS, without affecting other normal CNS tissues in order to avoid further damages. The use of nanoscale materials is expected to provide unique opportunities to: (i) improve drug solubility and bioavailability; (ii) enable targeted delivery and controlled release and, consequently, (iii) increase effective routes of administration and (iv) reduce toxicity. Interestingly, not only can nanovectors (extracellular and artificial vesicle nanoparticles) act as carriers of relevant molecules, but they can also trigger an immunomodulatory effect [13]. Indeed, variations in the chemical composition, size, and shape of the nanovector have a different impact on the immune target and immune response that could be even more significant in the context of autoimmune diseases [13]. In MS, the use of nanovectors has been investigated as drug delivery systems and as vectors for antigen-specific immunomodulation.

## 3. Extracellular Vesicles

**Figure 1:**
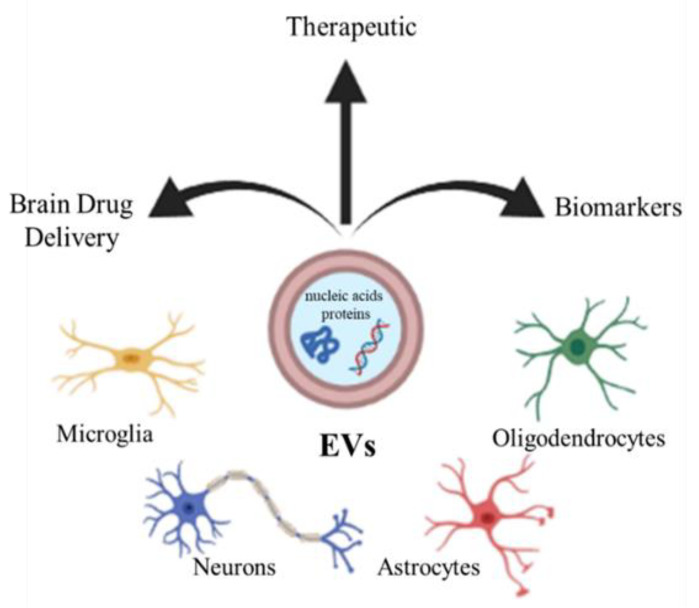
CNS cells (microglia, neurons, astrocytes, oligodendrocytes) release extracellular vesicles (EVs), which can be used as biomarkers, therapy, and drug vectors to the brain.

Extracellular vesicles (EVs) are an umbrella term for the small submicrometer-sized particles composed of lipid membranes released by the cells. Depending on their size, EVs are usually subdivided into exosomes, small particles with a diameter of 10–100 nm, microvesicles, larger vesicles whose diameter is in the range 100–1000 nm, and apoptotic bodies (>1000 nm) [14,15,16]. EVs are released by the different neural cells, including neurons, oligodendrocytes, astrocytes, and microglia [17,18]. It has been reported that EVs can deliver different kinds of molecules, such as nucleic acids and proteins, which often influence the phenotype of recipient cells [19,20,21]. The EVs play a role in several physiological processes in the CNS, such as development, myelination, regeneration, synaptic activity, and could be involved in neuropathology or, conversely, in regeneration and repair, providing protection against injury or promoting the illness [22]. Due to their content, EVs could therefore constitute a clinically important biomarkers for neurodegenerative diseases [23], or by constituting a physiological intercellular communication system, they can also represent candidates for therapeutic use, enclosing regulatory molecules [24]. Finally, EVs could be considered as carriers for specific brain drug delivery of small therapeutic molecules to contrast neurodegenerative disorders [24] (Figure 1).

**Figure 2:**
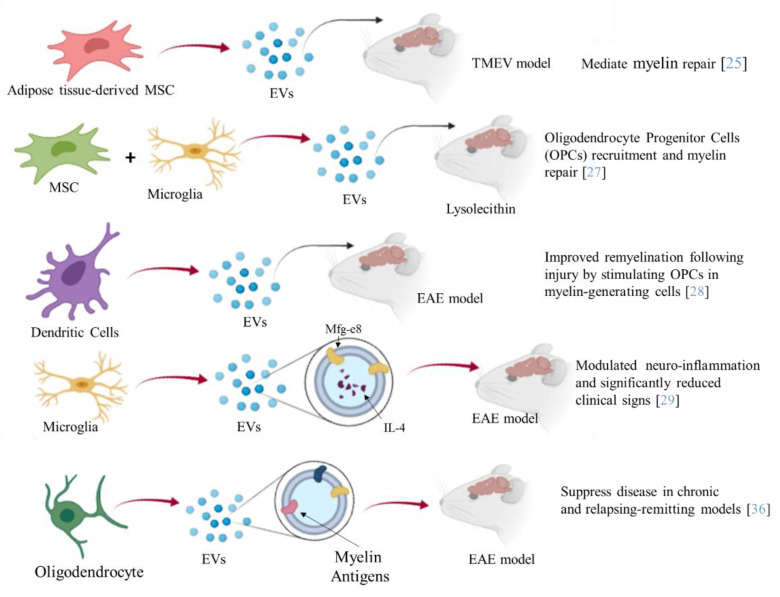
The picture summarizes the different aspects of the therapeutic potential of EVs in MS. EVs released from different cells, engineered or not, have been administered to different mouse models of MS. A reduction of the demyelination area, inflammation degree, and severity of MS symptoms have been observed.

Furthermore, EVs such as exosomes present good biocompatibility, low immunogenicity, and can easily pass through the BBB. Several studies have shown the therapeutic potential of EVs from different cellular sources against MS [25] (Figure 2). Administration of EVs derived from human adipose tissue-derived mesenchymal stem cells might mediate repair mechanisms in CNS damage and promote recovery in Theiler’s murine encephalomyelitis virus (TMEV)-induced demyelinating disease, a progressive model of MS [25]. Microglial cells are a component of the innate immune system within the CNS. Microglia participate in both myelin injury and remyelination during MS. During chronic inflammation, activated microglial cells can also participate in tissue destruction through antigen presentation and release of pro-inflammatory factors. Experiments on EAE have also revealed correlations between microglial and macrophage activation and the disease as their depletion or inactivation resulted in a delay of the disease onset along with decreased severity of clinical symptoms [26]. Lombardi and collaborators showed that EVs released by pro-inflammatory microglia blocked remyelination, whereas EVs produced by microglia co-cultured with immunosuppressive mesenchymal stem cells promoted Oligodendrocyte Progenitor Cells (OPCs) recruitment and myelin repair [27]. The authors proposed that the cargo, carried by EVs, has an effect on the inhibition of the OPCs differentiation, while EVs surface lipids have been identified as important factors to promote promyelinating action. In particular the sphingosine 1 phosphate (S1P) in EVs has been revealed as the key molecule promoting OPCs migration, the first step in the remyelination process [27]. Moreover, dendritic cells-derived exosomes administered to the brain markedly enhanced myelination and improved remyelination after injury by stimulating OPCs in myelin-generating cells [28]. In particular, the authors demonstrated that IFNγ-stimulated dendritic cells (DCs) released exosomes containing microRNAs that can increase basal myelination, reduce oxidative stress, and improve remyelination in lysolecithin-induced acute demyelination [28].

Moreover, engineered EVs may represent a biological drug delivery tool able to deliver simultaneously different multiple functional molecules to treat neuroinflammatory diseases. BV-2 murine microglial cells, have been engineered to release EVs, containing the Lactadherin (Mfg-e8) on the surface to target phagocytes and the cytokine IL-4 as anti-inflammatory agent [29].

A single injection of 10^7^ IL-4 + Mfg-e8 + EVs into the cisterna magna modulated neuro-inflammation and significantly reduced clinical signs of multiple sclerosis, in the EAE mouse model [29]. Mesenchymal stem cell (MSC) exosomes covalently conjugated with high-affinity aptamer toward myelin were administrated to EAE. The results indicated a robust suppression of the inflammatory response as well as a reduction of the demyelination lesion, resulting in reduced severity of the disease [30]. MSC-derived exosomes loaded with TGF-β, PD-L1, and Gal-1 were able to inhibit the autoreactive lymphocyte activation and proliferation by transferring these tolerogenic molecules. This was also due to the promotion of CD4^+^ CD25^+^ Foxp3^+^ Treg generation and apoptotic activity towards activated T cells [31]. Therapeutic effects of EL-4 (murine tumor cell line) exosomes loaded with curcumin (Exo-Cur) have been demonstrated in lipopolysaccharide (LPS)-induced brain inflammation and MOG-induced EAE [32]. In particular, by intranasal administration, Exo-Cur resulted in the rapid delivery of the encapsulated drug to the brain, by microglial cells absorption, thus preventing inflammation [32]. Curcumin is a known anti-inflammatory molecule with effects on numerous targets, through inhibition of interleukin 17 in different cell types [33]. EVs obtained from DCs overexpressing TGF-β1 in the membrane were obtained (TGF-β1-EVs) by Yu and collaborators [34]. The TGF-β1-EVs possessed immunosuppressive capacity and inhibited the development and progression of MS in a mouse model [34].

Moreover, EVs have been proposed to constitute an effective tool for the induction of immunetolerance [35]. In particular, intravenous injection of oligodendrocyte-derived extracellular vesicles that contain most, or possibly all, relevant myelin antigens (Ags), had the ability to induce Ag-specific tolerance and suppress disease in chronic and Relapsing-Remitting EAE models (RR-EAE) [36].

However, a prerequisite for EVs clinical applications is a standardized approach. In specific, it is urgent to improve their safety, the source (culture of patient cells), the extraction protocols, the reproducibility, and the characterization processes. In fact, the content and composition of EVs released from cells depend on the physiological status of the latter. All this anticipates intensive research in the field over the next years, and novel approaches to efficiently produce EVs are needed. Issues with yield and heterogeneity have hindered the clinical use of EVs [37]. Physical and mechanical processes, such as nitrogen cavitation, sonication, and extrusion, were used to force the production of EVs-like cell-derived vesicles (mimetic-EVs), with a production efficiency higher that that recorded for naturally secreted EVs [38]. To date, in the field of biomedical research related to MS, an EVs-like product with potential therapeutic applications does not exist. Indeed, a search in the Knowledge Network for “Extracellular vesicles-like and multiple sclerosis” or “Extracellular vesicles-mimetic and multiple sclerosis” (pubmed.gov) yielded no results.

In conclusion, we have described recent strategies on the use of EVs as potential therapy for MS. They have the advantage of being completely compatible with their host, crossing the physiological barriers, having a specific target, and transporting molecules with therapeutic potential. The heterogeneity of MS disease, the lack of adequate standardization in the isolation process of EVs, the need to isolate EVs from autologous cells, and the variety and variability of their content, which mirrors the cellular physiological state, make it difficult to translate the EVs in clinical practice. To overcome these limits, researchers could improve the methodology for the isolation and quantization of EVs and use bioengineered techniques to control and modify their cellular content.

## 4. Artificial Vesicles

With the aim of mimicking the characteristics of natural vesicles, the researchers focused on the fabrication of artificial vesicles (Art-Vs), which consist of an aqueous core encapsulated by a lipid bilayer (artificial membrane). The membrane-like structure is capable of loading lipid-soluble drugs while the hydrophilic nuclei have the ability to encapsulate water-soluble drugs [39].

**Figure 3:**
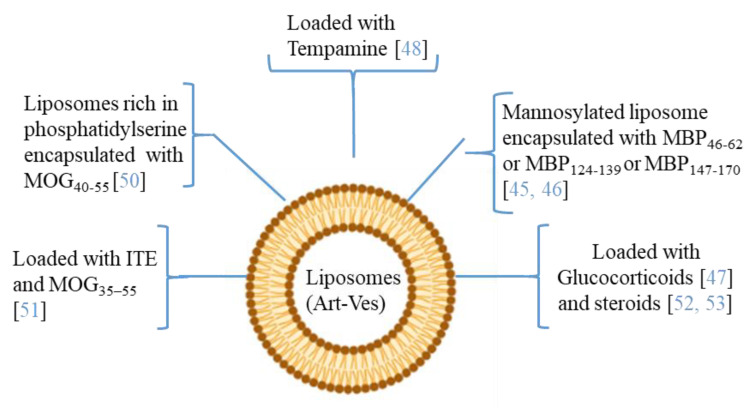
The picture illustrates some researches in which the therapeutic potential of liposomes/artificial vesicles loaded with different molecules, antioxidant, anti-inflammatory (immunosuppressive effect), myelin antigens (immunotolerance effect), or in combination, as a potential therapeutic approach in MS.

Moreover, since MS affects the CNS, delivering therapeutic agents across the BBB is an important challenge. The ability to modify both the chemical and physical characteristics (dimensions, shape, polydispersity, surface, stability, and loading capacity) of the Art-Vs allows different types of biomedical applications. Liposomes are the first type of Art-Vs investigated. Liposomes protect loaded molecules from degradation, and release them into specific target cells [40]. Moreover, to increase the BBB permeability and the concentration of the drug in CNS lesions, cell-penetrating peptides (Trans-acting Activator of Transcription-TAT), were introduced inside the liposomes [41]. Moreover, liposomes are approved by the Food and Drug Administration (FDA) as therapeutic agents for drug delivery due to their safety and tolerability [42]. Several data have indicated the therapeutic potential of liposomes in MS (Figure 3). In particular, many of the applications with liposomes concern the encapsulation of antigenic myelin peptides in order to induce an immune tolerance effect. The mechanisms of induction of tolerance can lead to the induction of DCs and immunosuppressive macrophages, to the reduction of CD4+ T helper 1 and 17 lymphocytes, and to the induction of regulatory T (Treg) cells [43,44]. 

In the MS application, MBP fragments encapsulated in mannosylated liposomes suppressed the encephalomyelitis autoimmune, in EAE rodent model of MS, reducing the severity of the first attack and facilitating recovery from the acute state [45]. The same authors studied the role of the polypeptide fragments [MBP46-62 (GH17), MBP124-139 (GK16), and MBP147-170 (QR24) peptides] encapsulated in mannosylated liposomes in the release of cytokines and activation of immune cells from MS patients and healthy donors. They demonstrated that GH17 rebalances T cells to being CD8^+^ and induces the production of IL-10 anti-inflammatory cytokine that may contribute to the improvement of the first disease attack. By contrast, QR24 and GK16, which are mainly effective in preventing a second wave of exacerbation of the disease, induce the release of pro-inflammatory cytokines, shifting the CD4/CD8 ratio to CD4 T cells and promoting the proliferation of CD4^+^ CD25^+^ lymphocytes, which are important for maintaining further immune tolerance [46]. More recently, nanosterically stabilized liposomes (nSSL, approved by the FDA for anticancer therapy) loaded with glucocorticoids have shown therapeutic efficacy in PLP-induced EAE. In animals treated with nSSL, recovery from acute disease was also faster than with Betaferon and GA [47]. Furthermore, administration of nSSL loaded Tempamine, in PLP SJL and C57/Bl6 MOG in EAE mice improved the acute and chronic phase of the disease in terms of clinical score and number of inflammatory infiltrates [48,49]. Pujol-Autonell and collaborators prepared liposomes rich in phosphatidylserine (PS) (PS-liposomes), a component present in apoptotic cells modulating the immune responses. PS is recognized by dendritic cell receptors, allowing the encapsulated autoantigen to be presented, inducing an immunotolerant effect. PS liposomes were loaded with the MOG40-55 peptide (MS autoantigen) (PSMOG liposomes) and tested in an EAE mouse model. DCs efficiently captured PSMOG liposomes, inducing a tolerogenic phenotype, ceasing the autoimmune reaction [50]. Kenison and collaborators used nanoliposomes NLPs to codeliver the tolerogenic aryl hydrocarbon receptor (AhR) ligand, 2-10H-indole-30-carbonyl)-thiazole-4-carboxylic acid methyl ester) (ITE) and the disease-specific peptide antigens (MOG35–55). The aryl hydrocarbon receptor (AhR) is a ligand-activated transcription factor that plays a role in modulating the immune response. NLPs with ITE and MOG35–55 induce antigen-specific tolerance and suppress disease in three preclinical mouse models of MS [51]. Prednisolone and methylprednisolone (steroids) encapsulated in PEGylated liposomes have improved clinical parameters in EAE, with respect to free drugs [52,53].

## 5. Nanoparticles

Nanotechnology approaches have been actively investigated for the development of new therapies for MS [54]. Nanoparticles (NPs) can employ a wide range of materials, including metals, synthetic and natural polymers. Engineered materials at the nanoscale are expected to provide unique opportunities to improve the stability, solubility, and bioavailability of encapsulated molecules, allowing targeted delivery and controlled release, promoting transport across physiological barriers, prolonging half-life, improving the routes of administration, and increasing safety and efficacy of the treatments [55]. The emergence of NP-based therapies for the induction of antigen-specific immune tolerance holds considerable promises for the future of the immunotherapy. Hunter and collaborators developed biodegradable poly (lactic-coglycolic acid) (PLGA) nanoparticles, which carry myelin antigens (Ags), capable of inducing robust tolerance and long-term comprehensive disease protection in the RR-EAE mouse model [56]. This was confirmed in further studies where Ag-polymer-PLGA applied to EAE mice in vivo induced the regulation of DCs and the following regulatory Th and T cells (Treg) [57]. Moreover, multifunctional systems that combine the delivery of antigens with immunomodulatory drugs can be used to turn the immune response more specifically and effectively. Subcutaneous administration of PLGA nanoparticles containing MOG35-55 and interleukin-10 (IL-10) showed a reduction in disease severity in EAE MS model [58]. PLGA nanoparticles loaded with PLP139–151 peptide and rapamycin, an immunosuppressive compound, through an intravenous and/or subcutaneous administration led to complete inhibition of disease relapses [59]. PLGA nanoparticles encapsulated with Ags and rapamycin in EAE animals demonstrated that, by interacting with APCs, including macrophages, they induced the proliferation of Ags-specific Tregs, and inhibited transgenic T cells. This treatment would be beneficial for the treatment or recovery of relapsed EAE [60]. Moreover, acetalated-dextran-NPs loaded with MOG and dexamethasone showed a therapeutic effect [61]. Montes-Cobos et al. designed hybrid inorganic-organic NPs that exhibited strong activity against human macrophages. They reported that these NPs were able to deliver glucocorticoids specifically to macrophages in the MS model while preserving their activity [62]. Gold nanoparticles loaded with MOG35–55 and ITE with respect to MOG-loaded particles were able to induce the functional regulatory T-cells in the MS animal model more efficiently [63]. Solid lipid nanoparticles (SLNs) are promising carriers, as they can pass the BBB and deliver therapeutic biomolecules to the brain, and have already been used for various neurological conditions [64].

**Figure 4:**
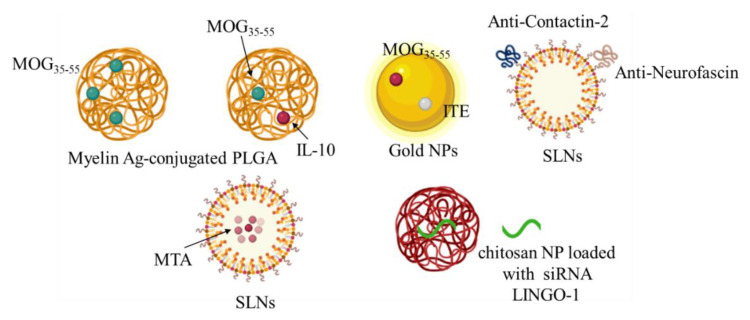
The picture summarizes the results of the therapeutic potential of different types of nanoparticles (NPs) loaded with different molecules, anti-inflammatory molecules and/or myelin antigens (immunotolerance effect), or functionalized (to increase a specific target) for a potential therapeutic approach against MS.

Oral administration of SLNs loaded with MTA (methylthioadenosine) in the cuprizone-induced demyelination model has been investigated [65]. Pharmacokinetic and pharmacodynamic studies have provided evidence of improved bioavailability and a longer biological half-life of the SLN-loaded MTA. The authors did not report evidence of the presence of SLN-MTA in the brain, but they demonstrated its effect in inducing remyelination of neurons [65]. Oral administration of DMF-loaded nanolipidic carriers coated with vitamins, tocopherol acetate cholecalciferol and retinol acetate, improved the clinical status (locomotor activity, coordination, and balance) and remyelination in a cuprizone-induced demyelination model [66]. Moreover, in order to design more efficient nano-carriers for MS treatment, the modified surface of PEGylated SLNs was prepared with anti-Contactin-2 or anti-Neurofascin, two antigens located in the Ranvier node. Brain uptake results demonstrated in the MS model mouse a greater brain uptake than that obtained with naked SLNs [67]. Using a similar strategy, PLGA NPs loaded with leukemia inhibiting factor (LIF) and functionalized with chondroitin sulfate NG2 proteoglycan antibodies were able to target oligodendrocyte precursor cells (OPC), inducing remyelination [68]. Administration of chitosan NP loaded with siRNA LINGO-1 (a protein suppressing myelination and axonal regeneration) in the mouse model of demyelination (ethidium bromide treatment), induced the downregulation of LINGO-1 associated with increased expression of MBP and lower levels of caspase-3 activity, and showed neuroprotection and remyelination effects [69] (Figure 4).

Liposomes and nanoparticles permit to solve the issues of yield and purity, which are typical limitations encountered by handling natural vesicles extracted from biological sources [70]. They have the advantage of being easily synthesized with the appropriate chemical-physical properties (size, charge, shape, polydispersity, stability) and functionalized with specific ligands, depending on the target to be reached. Furthermore, they can be loaded with multiple molecules that act on different aspects of a complex disease such as MS.

## 6. Conclusions and Future Developments

MS is a complex pathology with different players. In addition to the peripheral (T and B lymphocytes) and central (microglia) immune cells, the oligodendrocytes (remyelination) and neuron cells (degeneration) are also involved. This scenario is further complicated by the presence of physiological barriers, such as BBB, which hinder the route of the drug to the CNS. The set of scientific findings illustrated in this review highlights the high potential of nanotechnology for the development of new targeted therapies in MS. In the future, a deeper and broader synergy in the fields of materials science, bioengineering, biology, medicine, and drug discovery will certainly enable a more applicative use of nanotechnology in the treatment of MS. Moreover, the design of a delivery system, or the synergy of different systems, acting on all the involved actors would have a greater effect to modulate inflammation, central and peripheral, but also to activate the processes of remyelination and neuroprotection.
